# Screening Referable Diabetic Retinopathy Using a Semi-automated Deep Learning Algorithm Assisted Approach

**DOI:** 10.3389/fmed.2021.740987

**Published:** 2021-11-25

**Authors:** Yueye Wang, Danli Shi, Zachary Tan, Yong Niu, Yu Jiang, Ruilin Xiong, Guankai Peng, Mingguang He

**Affiliations:** ^1^State Key Laboratory of Ophthalmology, Zhongshan Ophthalmic Center, Sun Yat-sen University, Guangzhou, China; ^2^Centre for Eye Research Australia, University of Melbourne, Melbourne, VIC, Australia; ^3^Department of Ophthalmology, Guangzhou No. 11 People's Hospital, Guangzhou, China; ^4^Guangzhou Vision Tech Medical Technology Co. Ltd., Guangzhou, China; ^5^Department of Ophthalmology, Guangdong Eye Institute, Guangdong Provincial People's Hospital, Guangdong Academy of Medical Sciences, Guangzhou, China; ^6^Centre for Eye Research Australia, Royal Victorian Eye and Ear Hospital, Melbourne, VIC, Australia

**Keywords:** diabetic retinopathy, artificial intelligence, screening, cost-saving analysis, deep learning

## Abstract

**Purpose:** To assess the accuracy and efficacy of a semi-automated deep learning algorithm (DLA) assisted approach to detect vision-threatening diabetic retinopathy (DR).

**Methods:** We developed a two-step semi-automated DLA-assisted approach to grade fundus photographs for vision-threatening referable DR. Study images were obtained from the Lingtou Cohort Study, and captured at participant enrollment in 2009–2010 (“baseline images”) and annual follow-up between 2011 and 2017. To begin, a validated DLA automatically graded baseline images for referable DR and classified them as positive, negative, or ungradable. Following, each positive image, all other available images from patients who had a positive image, and a 5% random sample of all negative images were selected and regraded by trained human graders. A reference standard diagnosis was assigned once all graders achieved consistent grading outcomes or with a senior ophthalmologist's final diagnosis. The semi-automated DLA assisted approach combined initial DLA screening and subsequent human grading for images identified as high-risk. This approach was further validated within the follow-up image datasets and its time and economic costs evaluated against fully human grading.

**Results:** For evaluation of baseline images, a total of 33,115 images were included and automatically graded by the DLA. 2,604 images (480 positive results, 624 available other images from participants with a positive result, and 1500 random negative samples) were selected and regraded by graders. The DLA achieved an area under the receiver operating characteristic curve (AUC), sensitivity, specificity, and accuracy of 0.953, 0.970, 0.879, and 88.6%, respectively. In further validation within the follow-up image datasets, a total of 88,363 images were graded using this semi-automated approach and human grading was performed on 8975 selected images. The DLA achieved an AUC, sensitivity, and specificity of 0.914, 0.852, 0.853, respectively. Compared against fully human grading, the semi-automated DLA-assisted approach achieved an estimated 75.6% time and 90.1% economic cost saving.

**Conclusions:** The DLA described in this study was able to achieve high accuracy, sensitivity, and specificity in grading fundus images for referable DR. Validated against long-term follow-up datasets, a semi-automated DLA-assisted approach was able to accurately identify suspect cases, and minimize misdiagnosis whilst balancing safety, time, and economic cost.

## Introduction

Along with rapid increases in the prevalence of diabetes worldwide, growing numbers of patients are at risk of developing vision-threatening diabetic retinopathy (DR) that affects quality of life. DR has been reported in 103.1 million patients in 2020 and is estimated to affect 160.5 million in 2045 (1). Early detection and timely intervention are critical to prevent DR-related severe visual loss (2).

Regular screening is an effective strategy to identify DR patients amongst a large population, and those suitable for referral to ophthalmologists for treatment (3). However, annual screening as recommended by guidelines is poorly adhered to (4–6), especially in low- and middle- income regions (7). Given the uneven distribution of medical resources and retinal specialists in many countries, new methods and technologies are required to improve upon current screening strategies (8).

Automating the detection and classification of eye disease has received growing attention as a means to increase access to screening. Deep learning techniques are able to automatically capture and learn the most predictive features for classification from large training datasets (9). With no need to specify rules explicitly, deep learning algorithm (DLA)-based artificial intelligence AI systems have demonstrated promising accuracy and efficacy in detecting vision-threatening DR from fundus photographs (10–14), and may represent a cost-effective alternative to human grading. The implementation of these algorithms has the promise to enable more affordable, rapid, and consistent DR diagnosis.

Although now described extensively in the literature, the clinical application and translation of this AI technology remain limited. Researchers have made efforts to validate AI in clinical practice (15), revealing multiple socio-environmental factors that restrict the accuracy and adoption of this new technology (16). Clinical workflows present complex challenges including potential ethical, bias and generalizability limitations, and medicolegal implications when integrating these tools into existing clinical pathways (17). Further, the applicability and consistency of AI systems in detecting longitudinal change in single individuals has also not been evaluated.

As the clinical implementation of AI is challenging and underexplored, we thus conducted this study to integrate this technology into a research image dataset for the screening of vision-threatening referable DR. We tested a previously established DLA system in digital fundus photographs collected from the Lingtou Eye Cohort Study, which consisted of non-mydriatic fundus images routinely captured at annual health screening within primary care centers for enrolled general Chinese participants. In addition, to achieve improved screening performance, we developed a novel semi-automated DLA-assisted approach that combined both AI and human grading procedures, and tested its performance within longitudinal datasets. Lastly, to evaluate cost-savings, time and economic outlays for this semi-automated approach were compared against fully human grading alone (Supplementary Figure 1).

## Materials and Methods

### Study Design

The main aim of the study was to validate the screening performance of a previously established DLA against manual grading as a reference standard in a research image dataset. We then developed a novel semi-automated DLA-assisted workflow that integrated this DLA into a manual grading workflow and evaluated potential time and economic cost savings.

To test DLA performance in detecting vision-threatening referable DR (pre-proliferative DR or worse), a total of 33,115 fundus photographs captured at participant enrollment from the Lingtou Eye Cohort Study (“baseline images”) were included in this present study. The Lingtou Eye Cohort Study is an ongoing prospective cohort study that enrolled government employees attending the Guangzhou Government Servant Physical Check-up Center, Lingtou, China. At baseline recruitment in 2009–2010, a total of 4,939 participants were enrolled and all subsequently invited to take part in annual follow-up at primary care centers, including physical and ophthalmic examinations and health questionnaires. Detailed study methodology has been reported previously (18). Written informed consent was obtained from all participants.

### Collection of Fundus Photographs

Non-mydriatic standard digital fundus photographs were captured of each eye using a fundus camera (TRC-NW6S; Topcon, Tokyo, Japan) in two specified positions: centered on the optic disc (F1 image) and macular fovea (F2 image). Among 4,939 participants included at baseline, 39 did not receive fundus photography due to lack of cooperation, rejection of ophthalmic examination, amongst other reasons. Images from the remaining 4,900 participants were captured. During annual follow-up from 2011 to 2017, the numbers of participants with fundus photographs captured annually were 3,505, 3,325, 3,198, 3,104, 3,038, 2,873, and 2,579, respectively.

Original images included from baseline and follow-up were all jpeg files with a resolution of 2,000 × 1,980 pixels. Pre-processing was performed to normalize original images into an appropriate format for the DLA. Firstly, all images were clipped to 90% of its original size to remove the “boundary effect” and following, images were resized to a resolution of 299 × 299 pixels with red-green-blue (RGB) channels. Further, to eliminate potential noise in images, the local average color was subtracted and mapped to 50% gray.

### Development of Semi-automated DLA-Assisted Approach

Using an Inception-v3 convolutional neural network, we previously trained an AI-based DLA for the automated detection of referable DR. The Inception-v3 neural network structure for the DLA was trained from scratch with a mini-batch gradient descent size of 32 and Adam optimizer of a 0.002 learning rate (19). After image normalization, the input of each image was transformed into a standard format of 299 × 299 × 3, while outputs were warped into probability distributions (Supplementary Figure 2). A total of 71,043 retinal photographs (both F1 and F2 images) from different hospitals and clinics in China were used for training and internal validation. The formats of images used during development of the DLA were the same as those in this study. Detailed descriptions of this DLA have been reported in our previous study (19).

For these training and internal validation data, criteria used for grading DR originate from the National Health Service (NHS) (20). Images were categorized as R0 (no DR), R1 (background DR), R2 (pre-proliferative DR), and R3 (proliferative DR). Images categorized as R3 were further subcategorized as R3a for active disease, and R3s for stable disease. Images of poor quality or poor positioning were defined as ungradable. Vision-threatening referable DR was defined as either pre-proliferative or proliferative DR (R2, R3a, and R3s as per NHS guidelines). Detailed criteria for classification and fundus images for typical cases are shown in Supplementary Table 1 and Supplementary Figure 3.

Performance of DLA grading was validated using images from the Lingtou Cohort and compared against trained human grader results as reference. Manual grading was carried out by three trained graders and two licensed ophthalmologists. All graders were masked to participant diagnoses and baseline characteristics to minimize bias and ensure comprehensive validation of the DLA's initial diagnosis. If grading outcomes were not consistent among graders, images would be reviewed by an assigned senior ophthalmologist, with diagnoses assigned at this step considered conclusive. A reference standard grading would be assigned once all graders achieved consistent grading outcomes or with a senior ophthalmologist's final diagnosis.

Utilizing the DLA system we previously established, we carried out a two-step semi-automated DLA-assisted approach to screen images sourced from the cohort study. Firstly, using this DLA, all images captured at baseline and at follow-up were screened for referable DR and classified as positive, negative, or ungradable. Secondly, all images classified as positive by the DLA, and all other available images from the same patient (even if classified as negative or ungradable) would be selected and regraded by trained graders. In addition, images initially graded as negative by the DLA were randomly sampled for further trained grader review. Approximately 5% of images were randomly sampled and independently regraded by three trained graders. Finally, any images where there was disagreement between the DLA and human grader were manually reviewed, to investigate the causes that may underlie false DLA diagnoses.

### Cost-Saving Evaluation

We recorded the total working time and economic costs of grading for this semi-automated DLA-assisted approach and compared it to a fully human grading process. The cost-saving analysis were performed based on follow-up datasets with a total of 88,363 images.

The total working time for the semi-automated approach was calculated as the sum of time required for the DLA to grade all images, and time required for subsequent human grader review of flagged images. As only flagged images within the follow-up datasets were reviewed by human graders, the time-cost human graders would require to grade the full dataset was calculated by multiplying the average time required per image across the total number of images.

In the calculation of economic costs of grading, only costs of human grading (i.e., payment to human graders as remuneration for their time) were considered in the cost-saving evaluation of the semi-automated and fully manual grading approaches. DLA costs were not considered as the marginal cost of DLA operation is minimal, and the cost of DLA development was outside the scope of this study. Time and economic costs for grading per image were evaluated and compared across the fully DLA grading, semi-automated DLA-assisted grading, and fully human grading approaches.

### Statistical Analysis

To assess the validity of classification by the DLA; sensitivity, specificity, accuracy, and area under the receiver operating characteristic curve (AUC) with 95% confidence intervals (CI) were calculated and compared to human grading results. A weighted kappa (κW) statistic (with Cicchetti–Allison weighting) was used to compare agreement between the DLA and human grading.

Statistical analyses were performed using standard statistical software (R, v.4.0.4; Stata, v.15.1).

## Results

### Assessment of DLA and Human Grading

**Figure 1:**
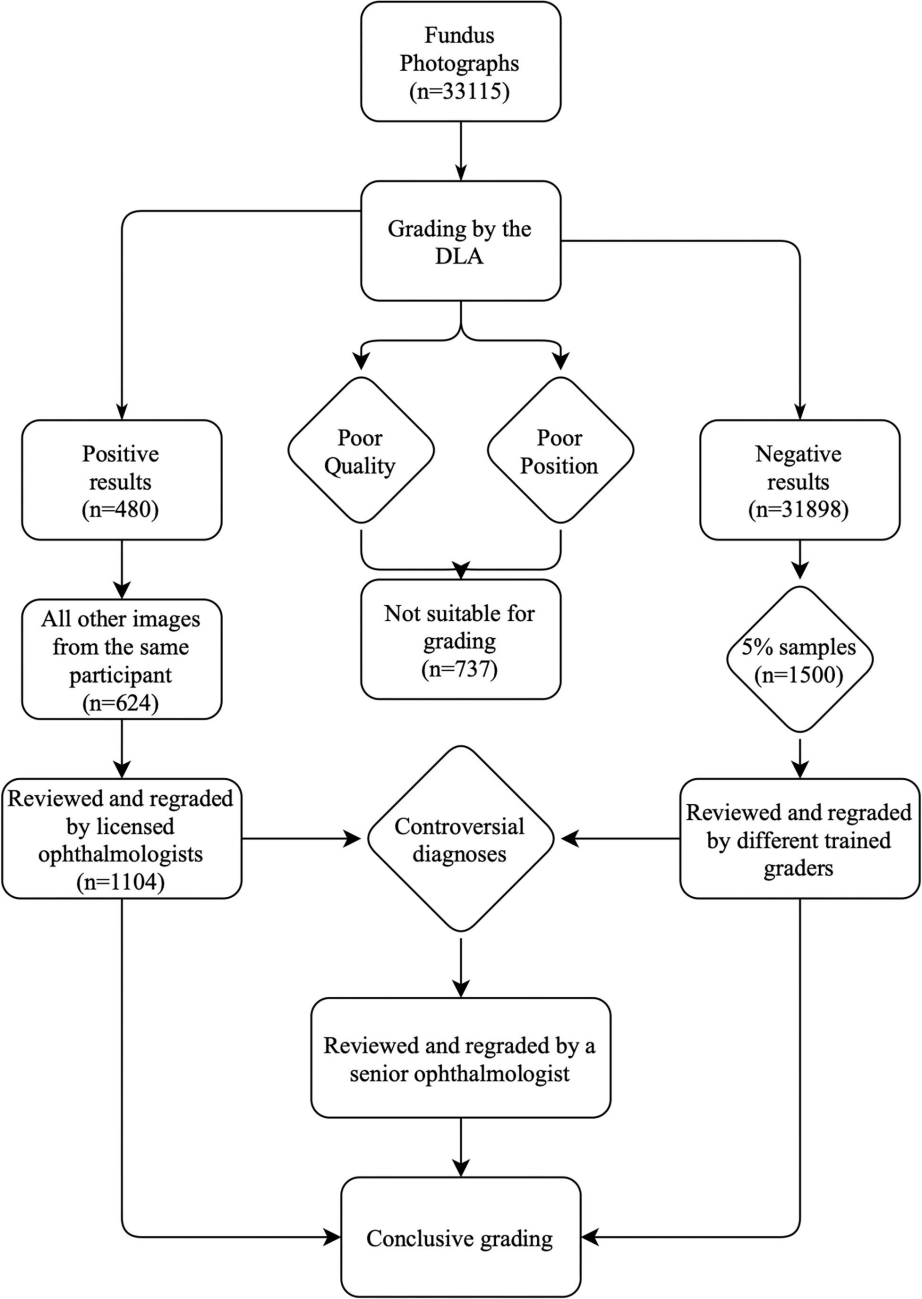
Grading workflow of semi-automated DLA-assisted detection of referable DR. DLA, deep learning algorithm; DR, diabetic retinopathy.

A total of 33,115 fundus photographs were captured at participant enrollment for the Lingtou Eye Cohort Study (“baseline images”) and screened for referable DR. Baseline clinical characteristics of included participants were shown in Table 1. Among these images, 737 (2.23%) were classified as ungradable due to poor quality or poor position, which may result from extremely small pupils or eye movement during image capture. A total of 32,378 images were conclusively graded by the DLA. For further human grading, images graded as positive by the DLA (*n* = 480, 1.45%), other images from participants with an image graded as positive by the DLA (*n* = 624, 1.88%), and randomly selected negative samples (*n* = 1500, 4.5%), were reviewed (Figure 1). After manually grading by trained human graders, 191 images were classified as true-positive (TP) and 2,117 as true-negative (TN), while 289 were classified as false-positive (FP) and 7 as false-negative (FN) (Table 2).

**Table 1:** Characteristics of baseline participants with fundus images.

**Characteristics**	**Baseline (2009–2010)**
No. of participants	4,900
Age (mean ± sd, yrs)	59.1 ± 8.78
Male (*n*, %)	2,846 (58.5%)
Smoke (*n*, %)	823 (16.9%)
Alcohol consumption (*n*, %)	2,545 (52.2%)
BMI (mean ± sd, Kg/m2)	24 ± 3.00
SBP (mmHg)	129 ± 18.0
DBP (mmHg)	75.0 ± 11.3
Fast blood glucose (mmol/L)	5.60 ± 1.17
Triglyceride (mmol/L)	1.75 ± 1.43
Cholesterol (mmol/L)	5.45 ± 0.95
Diabetes (*n*, %)	591 (12.0%)
Diabetes duration of diabetic patients (mean ± sd, yrs)	6.44 ± 6.01

*BMI, body mass index; SBP, systolic blood pressure; DBP, diastolic blood pressure*.

**Table 2:** Confusion matrix of DLA grading results for baseline images.

**No. of fundus images**		**Reference (human grading)**		
		**Positive**	**Negative**	**Total**
DLA grading	Positive	(TP) 191	(FP) 289	480
	Negative	(FN) 7	(TN) 2,117	2,124
	Total	198	2,124	2,604

*DLA, deep learning algorithm; TP, true-positive; FP, false-positive; FN, false-negative; TN, true-negative*.

**Figure 2:**
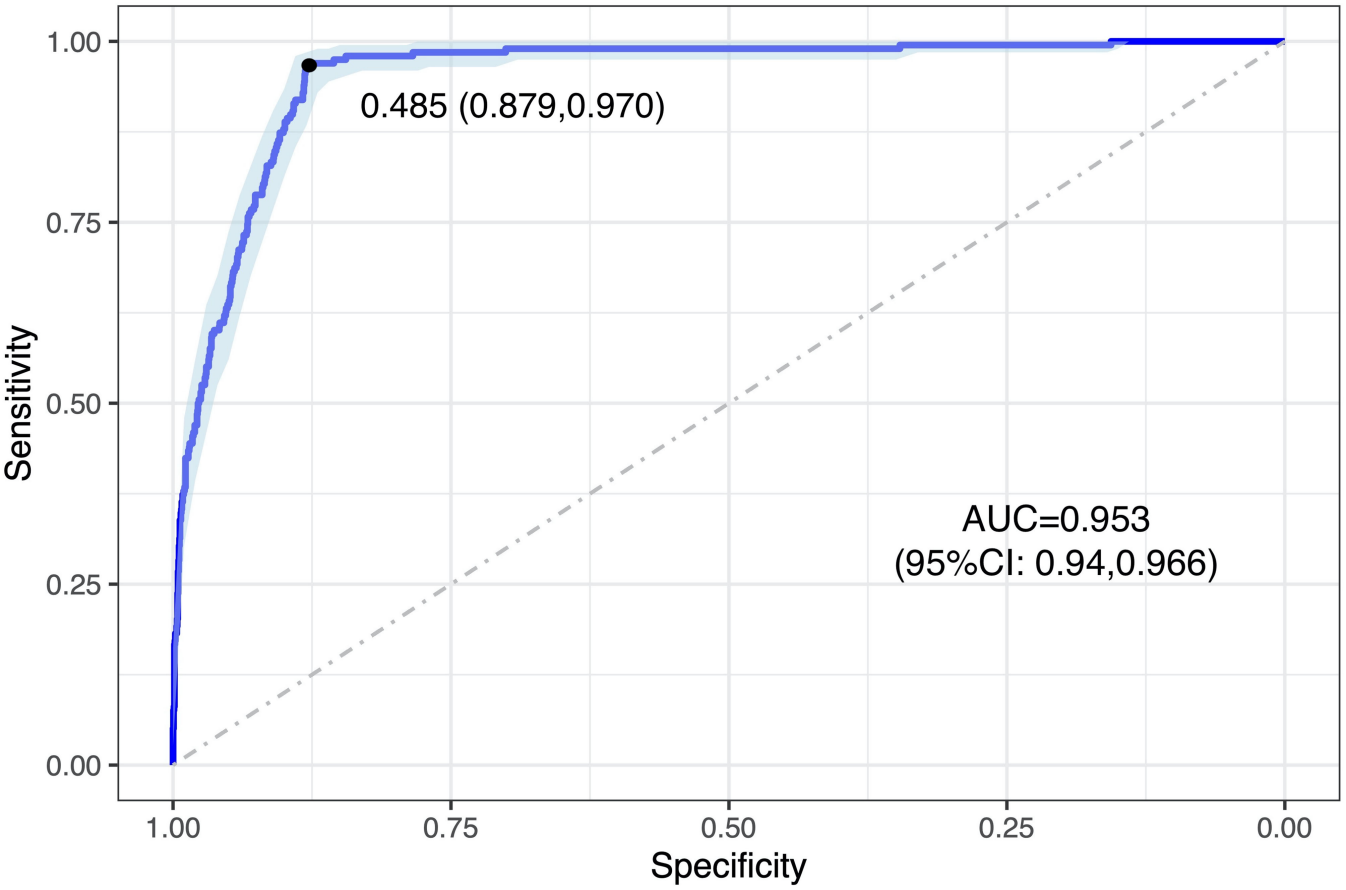
ROC curve of DLA grading in analyzing baseline images. The blue curve represents the model's trade-off, with the black dot marking the threshold point with an optimal cut-off value. This threshold point yields an optimal cut-off probability for having referable DR of 0.485, with a specificity and a sensitivity of 0.879 and 0.970, respectively. ROC, receiver operating characteristic; AUC, area under the receiver operating characteristic curve; DLA, deep learning algorithm.

As a result, 88.6% [(TP + TN)/total graded images] of the baseline images were correctly graded by the DLA compared to human grading. Agreement between the DLA and human grading was acceptable with a κW of 0.511 (95% CI: 0.465–0.557). Compared to human grading, the AUC, sensitivity, and specificity of the DLA were 0.953 (95% CI: 0.94–0.966), 0.970, and 0.879 respectively, with a cutoff point of 0.485 (Figure 2).

**Figure 3:**
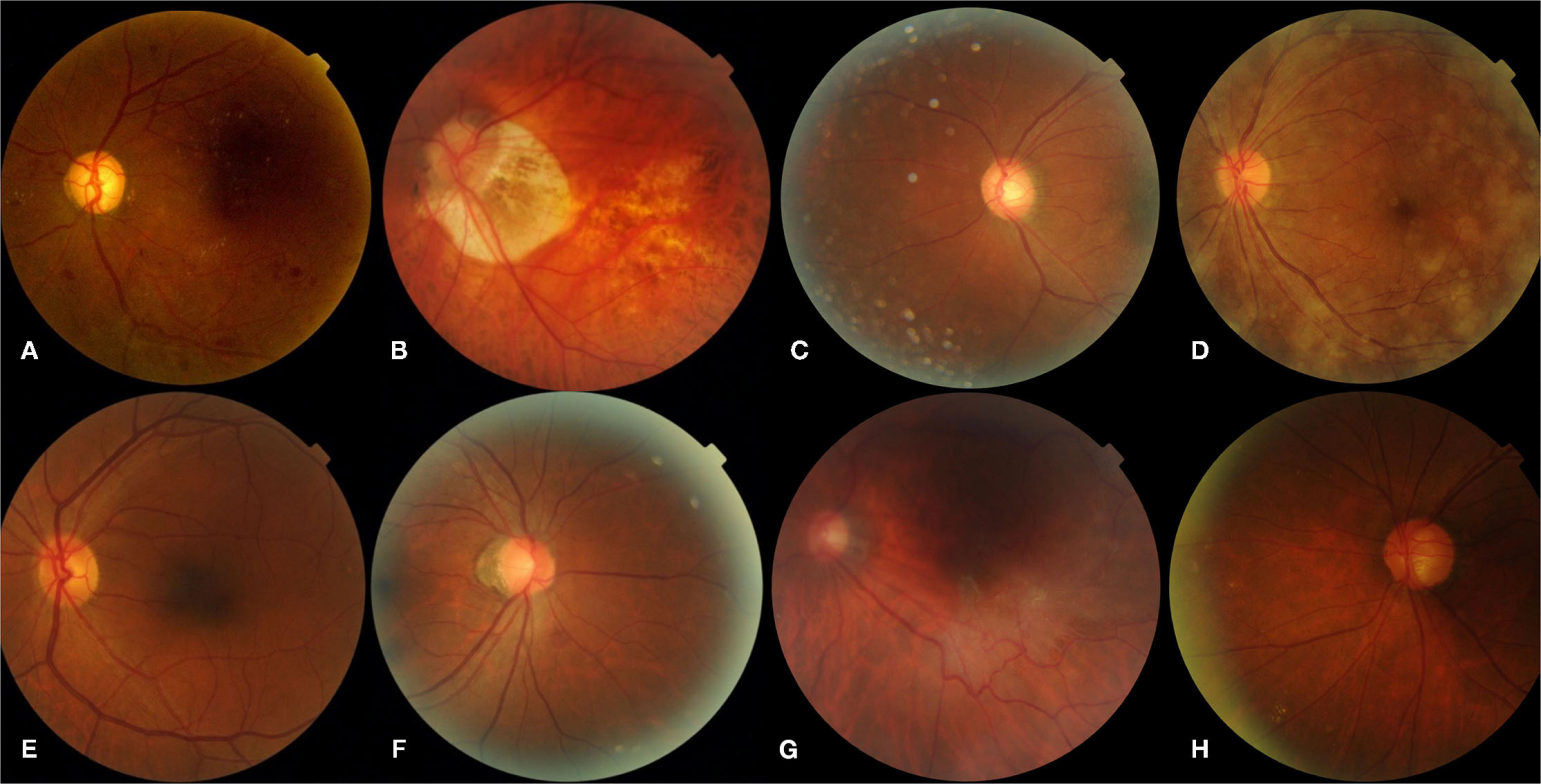
Representative sample of typical images classified by the DLA. **(A)** Represents a true-positive image, **(B–D)** represent typical false-positive images, **(E)** represents a true-negative image, **(F–H)** represent typical false-negative images. **(A)** Pre-proliferative DR (R2) with microaneurysms, multiple blot hemorrhages, hard exudates; **(B)** myopic retinopathy; **(E)** normal fundus with artifacts; **(D)** opaque refracting media; **(A,E)** normal fundus; **(F)** intra-retinal microvascular abnormalities; **(G)** questionable new vessels; **(H)** blurred peripheral retina exudates. DLA, deep learning algorithm.

The most common cause of false-positive cases (*n* = 289) was myopic retinopathy [*n* = 102 (35.29%)], associated with diffuse or patchy choroidal atrophy, while intraretinal microvascular abnormalities [*n* = 3 (42.86%)] were most commonly responsible for the false-negative cases (*n* = 7). Features and examples of typical cases classified by the DLA are shown in Table 3 and Figure 3.

**Table 3:** Features and numbers of DLA-classified false-positive and false-negative referable DR cases.

**Features**	**No**.	**Proportion**
False-positive images		
Myopic retinopathy	102	35.29%
Normal fundus with artifacts	80	27.68%
Opaque refracting media	32	11.07%
Age-related macular degeneration	28	9.69%
Retinal pigment changes	17	5.88%
Retinal atrophy	9	3.11%
Retinal vessel occlusion	8	2.77%
Background DR	2	0.69%
Others	11	3.81%
Total	289	100.00%
False-negative images		
Intraretinal microvascular abnormalities	3	42.86%
Blurred peripheral retina exudation	2	28.57%
Diabetic macular exudation	1	14.29%
Questionable new vessels	1	14.29%
Total	7	100.00%

*DLA, deep learning algorithm; DR, diabetic retinopathy*.

### Semi-automated DLA-Assisted Grading Approach Testing

To test the reproducibility and reliability of the semi-automated DLA-assisted grading approach, we applied this protocol to fundus images captured at annual Lingtou Eye Cohort Study follow-ups from 2011 to 2017. A total of 88,363 fundus photographs from these 7 years were obtained and graded. The percentage of ungradable images due to poor quality or positioning was on average 4.72% across the annual datasets. DLA performance across these years is shown in Table 3. As per the semi-automated study protocol, 8,975 images with either positive DLA grading or other images from participants with an image graded as positive by the DLA were selected and re-graded by human graders. Compared against human grading, the AUC, sensitivity, and specificity of the DLA across the different follow-up years were 0.914 (0.87–0.939), 0.852 (0.718–0.915), 0.853 (0.741–0.918) respectively, as shown in Table 4 and Supplementary Figure 4.

**Table 4:** DLA grading results across the baseline and 2011-2017 follow-up cohorts.

**Grading for referable DR**	**Baseline**	**2011**	**2012**	**2013**	**2014**	**2015**	**2016**	**2017**	**Total in follow-up**
No. of participants	4,900	3,505	3,325	3,198	3,104	3,038	2.873	2,579	-
No. of included images	33,115	13,927	13,590	13,089	12,929	12,303	11,749	10,776	88,363
Positive images (*n*, %)	480 (1.45%)	186 (1.34%)	185 (1.36%)	169 (1.29%)	182 (1.41%)	199 (1.62%)	196 (1.67%)	150 (1.39%)	1267 (1.43%)
Negative images (*n*, %)	31,898 (96.3%)	13,363 (96.0%)	12,992 (95.6%)	12,378 (94.6%)	11,834 (91.5%)	11,316 (92.0%)	10,998 (93.6%)	10,094 (93.7%)	82,975 (93.9%)
Ungradable images (*n*, %)	737 (2.23%)	378 (2.71%)	413 (3.04%)	542 (4.14%)	913 (7.06%)	788 (6.40%)	555 (4.72%)	532 (4.94%)	4121 (4.72%)
No. of images graded by human	2,604	1,628	1,419	1,355	1,333	1,179	1,099	962	8,975
Positive images by human grading	198	57	62	55	80	76	91	71	492
AUC	0.953	0.870	0.910	0.919	0.939	0.921	0.937	0.899	0.914
Sensitivity	0.970	0.903	0.855	0.873	0.887	0.816	0.915	0.718	0.852
Specificity	0.879	0.741	0.883	0.835	0.867	0.900	0.824	0.918	0.853
Accuracy of the DLA	0.886	0.921	0.913	0.916	0.923	0.896	0.904	0.918	0.913

*DLA, deep learning algorithm; DR, diabetic retinopathy; AUC, area under the receiver operating characteristic curve*.

For the fully automated DLA grading process, all 88,363 follow-up images were classified within 84.8 h, with an average grading time of 0.058 min per image. In the human grading process, three graders and two ophthalmologists combined required 55 h on the whole to grade a total of 8,975 flagged images, with an average grading time of 0.368 min per image. For this, human graders were paid a total of $1544 United States Dollars (USD) ($0.172 per image). The hypothesized time and economic cost that would be required for human graders to grade all follow-up images was calculated as ~543 h and $15,202 USD.

The total time required for semi-automated DLA-assisted analysis of the follow-up datasets was 139 h (0.09 min per image), and the total cost ~$1544 USD ($0.017 per image). This represented an estimated 75.6% time and 90.1% cost saving compared to entirely human grading of the same dataset. Comparison of the total and mean time and cost of the different grading protocols (fully automated DLA grading, human grading, and semi-automated DLA-assisted grading) are shown in Table 5.

**Table 5:** Comparison of time and cost for grading follow-up images between different grading procedures.

**Cost-saving analysis**	**Fully human grading**		**Fully automatic DLA grading** **(*n* = 88,363)**	**Semi-automated DLA-assisted grading** **(*n* = 88.363)**
	**For flagged images** **(*n* = 8975)**	**Hypothesized for whole dataset** **(*n* = 88,363)**		
Time for grading (h)	55	543	84.8	139
Mean time of grading per image (min)	0.368	0.368	0.058	0.094
Total cost of grading ($)	1,544	15,202	–	1,544
Mean cost of grading per image ($)	0.172	0.172	–	0.017

*DLA, deep learning algorithm*.

## Discussion

Although various AI-based algorithms have been developed for DR detection, implementation within clinical practice remains nascent and under investigation. In this study, we developed a semi-automated DLA-assisted approach to diagnose referable DR and validated it across 121,478 images collected longitudinally from a cohort study. This semi-automated DLA-assisted approach presented advantages in time and economic savings for grading, with combined DLA and human grading enabling accurate and efficient diagnoses.

With rapid advances in the development of AI technology for detecting DR, some studies have investigated the clinical application of AI-based grading and diagnosis (21, 22). Within limited samples, the performance of AI tools has been impressive. He et al. conducted a study of AI-based screening for DR amongst 889 diabetic patients at a Chinese community hospital, achieving high sensitivity (90.8%) and specificity (98.5%) in the detection of DR (23). Real-world performance may however be affected in larger populations. According to a recent multicenter study, 7 DR screening algorithms had significant variability in performance on real-world clinical data, with sensitivities varying from 50.98 to 85.90% (24). Shah et al. evaluated the performance of an AI algorithm in the detection of referable DR in a screening program with 2,680 diabetic patients, achieving 100% sensitivity and 82% specificity (25). These results however may not be ideal in a primary care screening setting given relatively low expected positive predictive value, which may result in individuals who are healthy or have background DR mistakenly flagged for referral (26). DR screening guidelines issued by Diabetes UK recommend a minimum of 95% specificity, which remains a challenging threshold for AI grading to achieve in large real-world settings (27). Within our 8-year longitudinal cohort study dataset, our DLA was not able to achieve the recommended 95% specificity standard. In light of the challenges of using AI to completely replace human grading, a more practical solution may be the integration of AI into existing DR screening workflows. The semi-automated DLA-assisted approach described in this present study may be more feasible in achieving the requisite accuracy.

To implement new AI diagnostic strategies within clinical practice, three different general models have been previously proposed: triage, replacement, and add-on (28). All these models have been trialed for DR screening in previous attempts. Tufail et al. trialed a replacement and add-on model for DR screening, where automated retinal image analysis served as either an alternative for human grading, or as a filter prior to manual grading (29). Within a real-world screening environment, both the replacement and add-on filter strategies achieved acceptable accuracy at a lower overall cost of grading compared to fully human grading. In triage models, AI tools are likely to be adopted as diagnostic decision-making supports, that generate immediate reports for qualified clinicians to review. One triage model that adopted AI in endocrinology and primary care settings within Australia reduced the workload for telemedicine graders by over 50% (17).

The semi-automated DLA-assisted approach described in this present study may be adopted within a triage model, and potentially reduce the workload of human graders. For instance, images in which the DLA has deemed to be highly likely to be negative may not require further review, enabling human graders to focus their review on images in which the pre-grading probability of DR is significantly higher. We were able to validate the accuracy of this semi-automated DLA-assisted approach within a general population with a significantly lower prevalence of DR (2), compared to previous validation studies that were performed in diabetic populations with a significantly higher existing prevalence of DR (30). This is an important contribution in validating the adoption of AI-based DR screening for general populations, including within primary care, where there is a very low pre-test probability of patients having referable DR. In addition, our findings validate the performance of a semi-automated DLA-assisted approach, where DLA graded negative images can safely not require further human grader review.

Despite requiring additional human grader review for some images, our semi-automated DLA-assisted approach achieved substantial cost savings compared to a fully human grading approach. Previous studies including the Singapore Epidemiology of Eye Diseases Study evaluated the cost-saving of two AI-based DR screening models, comparing a fully automated approach and a semi-automated approach (in which the AI served as a triage filter), against fully human grading (31). That study found the semi-automated approach to be the least expensive, achieving an estimated 20% saving in current annual screening costs if applied across the health system. Similarly, the semi-automated DLA-assisted approach described in this study may represent a significant cost saving compared to a fully human grading approach, particularly when applied to screening large general populations in primary care settings. These time and economic savings may be increasingly important going forward, as the requirement for grading and diabetic retinopathy screening increases with the growing population prevalence of diabetes. In this present study, we have shown that a semi-automated DLA approach may address some of these challenges.

The findings of this study must be considered in the context of its limitations. Firstly, the semi-automated DLA-assisted approach described in this study was only assessed within the Lingtou Cohort Study datasets, which is representative only for the primary care setting within this region. Further application in other settings requires caution. Secondly, ~5% of images were ungradable due to poor quality or poor positioning, which have may resulted in some positive DR cases being missed. Established standards for fundus photography should be strictly followed to minimize potential ungradable images. Thirdly, fundus images used in this study were two-field, non-stereoscopic images, which were easy and quick to capture in primary care clinics, but may have reduced DR detection compared to gold standard seven-field stereoscopic images. Additional studies may be required to better understand and optimize the means in which these diagnostic strategies are integrated into existing clinical practice workflows, optimizing for accuracy, cost, and accessibility.

In conclusion, the DLA described in this study was able to achieve high accuracy, sensitivity, and specificity in detecting vision-threatening referable DR. A semi-automated DLA-assisted approach that integrates initial automated DLA diagnosis and human grading for high-risk cases, was able to accurately identify suspect DR and avoid unnecessary review. Validated across multiple datasets, this approach minimized misdiagnosis whilst balancing safety, time, and cost. This approach has potential for adoption in increasing DR screening efficiency, enabling clinicians to meet the increasing eye care demands of rapidly growing global diabetes prevalence.

## Data Availability Statement

The raw data supporting the conclusions of this article will be made available by the authors, without undue reservation.

## Ethics Statement

The study was approved by the Ethics Committee of the Zhongshan Ophthalmic Center, Sun Yat-sen University, Guangzhou, and conducted in accordance with the Declaration of Helsinki. Written informed consent was obtained from all participants.

## Author Contributions

MH: study conception, design, and supervision. YW, DS, YN, YJ, and RX: acquisition, analysis, or interpretation. YW and ZT: drafting of the manuscript. MH, DS, ZT, and GP: critical revision of the manuscript for important intellectual content. YW, YJ, and RX: statistical analysis. YN and MH: administrative, technical, or material support. All authors contributed to the article and approved the submitted version.

## Funding

The present work was supported by the Fundamental Research Funds of the State Key Laboratory of Ophthalmology, Project of Investigation on Health Status of Employees in Financial Industry in Guangzhou, China (Z012014075), and Science and Technology Program of Guangzhou, China (202002020049). MH receives support from the University of Melbourne at Research Accelerator Program and the CERA Foundation. The Centre for Eye Research Australia receives Operational Infrastructure Support from the Victorian State Government. The sponsor or funding organization had no role in the design or conduct of this research.

## Conflict of Interest

GP is employed by Guangzhou Vision Tech Medical Technology Co. Ltd., Guangzhou, China. The remaining authors declare that the research was conducted in the absence of any commercial or financial relationships that could be construed as a potential conflict of interest.

## Publisher's Note

All claims expressed in this article are solely those of the authors and do not necessarily represent those of their affiliated organizations, or those of the publisher, the editors and the reviewers. Any product that may be evaluated in this article, or claim that may be made by its manufacturer, is not guaranteed or endorsed by the publisher.

## Abbreviations

DLA: deep learning algorithm
DR: diabetic retinopathy
AUC: area under the receiver operating characteristic curve
CBIA: computer-based image analysis
AI: artificial intelligence
NHS: National Health Service
CI: confidence intervals
κW: weighted kappa
USD: United States Dollars.
