# Supplementary material for: Screening Referable Diabetic Retinopathy Using a Semi-automated Deep Learning Algorithm Assisted Approach
Source: Front Med (Lausanne). 2021 Nov 25;8:740987. doi: 10.3389/fmed.2021.740987 (PMC8656222; doi:10.3389/fmed.2021.740987)
Supplement: Supplementary file 1 [file Data_Sheet_1.DOCX]

Supplementary Material

# Supplementary Figures and Tables

## Supplementary Figures

**Supplementary Figure 1.** **The proposed workflow to establish and validate the semi-automated DLA approach.**

Abbreviations: DLA, deep learning algorithm.


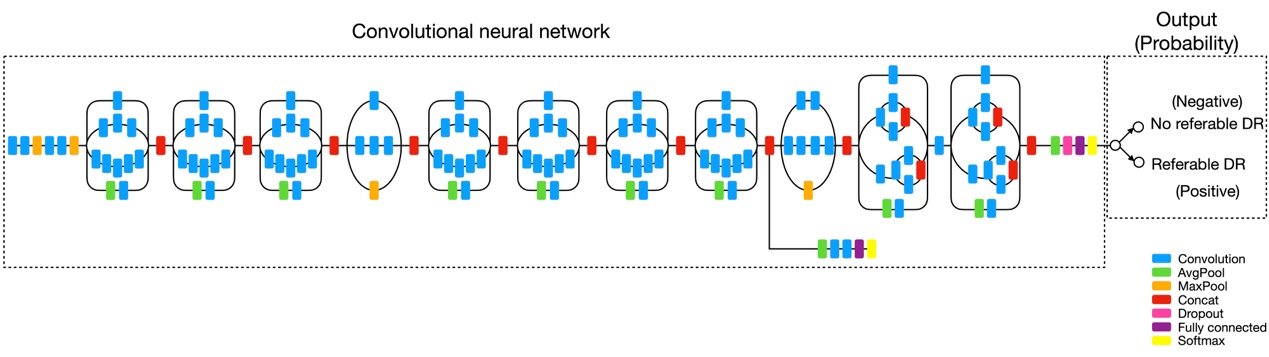


**Supplementary Figure 2. Structure of the Inception-v3 convolutional neural network for the DLA model to detect referable DR.**

The deep convolutional neural network model used in the DLA of this study. The data stream is from left to right. Firstly, images all images were clipped to 90% of its original size, and then normalized to a standard format for input of the Inception-v3 convolutional neural network as 299 × 299 × 3. Local average color would be subtracted and mapped to 50% grey to eliminate potential noise in images. The output for each gradable image would be a probability distribution of having referable DR, and further classified as negative (no referable DR) or positive (referable DR) based on the optimal cutoff.

Abbreviations: DLA, deep learning algorithm; DR, diabetic retinopathy.


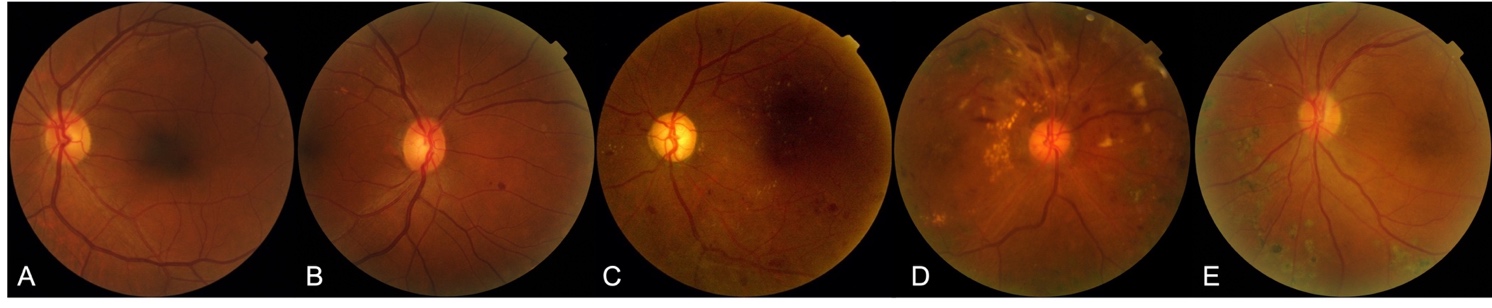


**Supplementary Figure 3. Typical images for different classifications of DR according to the NHS guidelines.**

A, R0, normal fundus; B, R1, background DR with retinal hemorrhage; C, R2, pre-proliferative DR with multiple blot hemorrhages; D, R3a, active proliferative DR with active fibrous proliferation; E, R3s, stable proliferative DR after peripheral retinal photocoagulation with laser scar.

Abbreviations: NHS, National Health Service; DR, diabetic retinopathy.


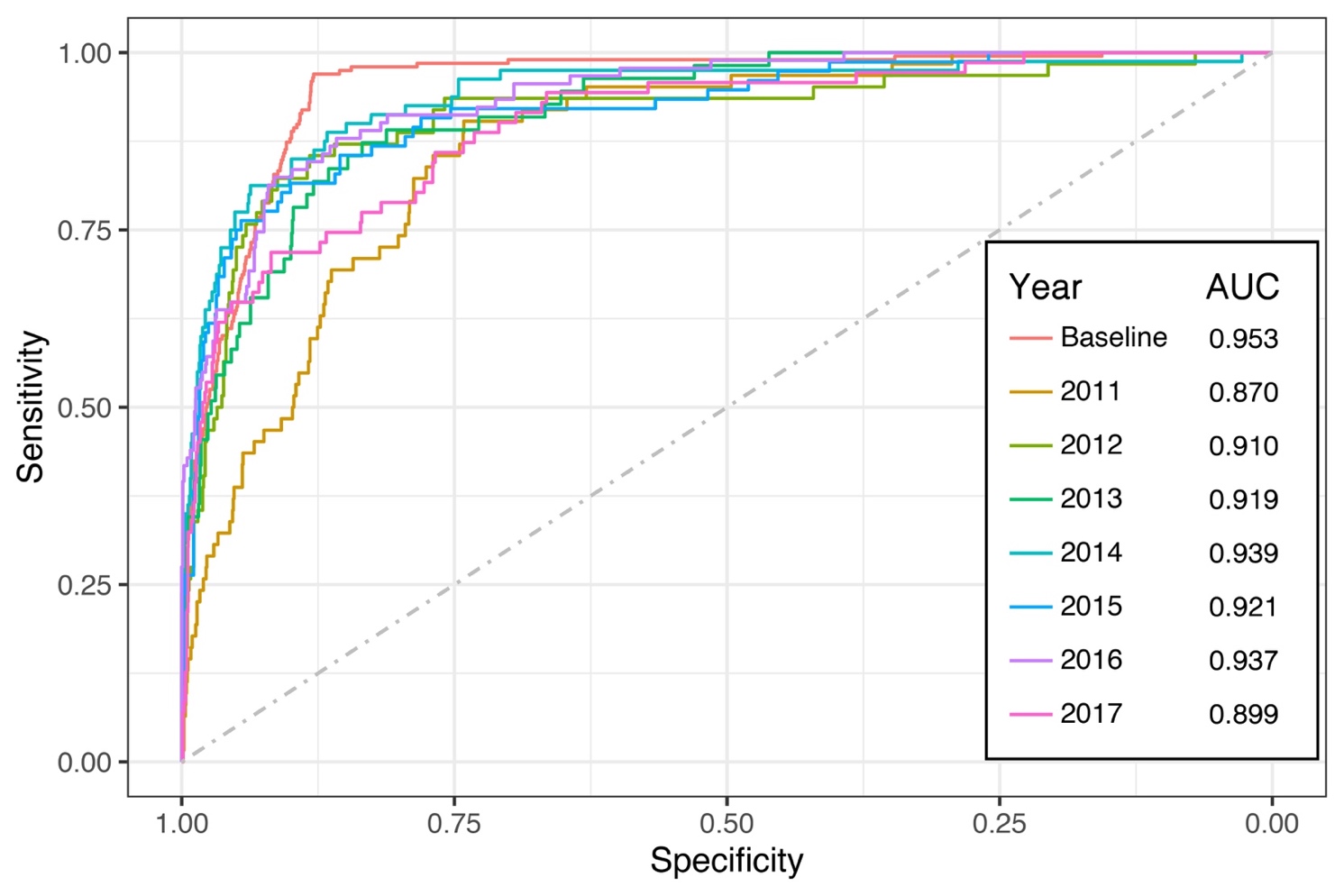
 **Supplementary Figure 4.** **The ROC curve of DLA grading in analyzing images from baseline to 2017.** The AUC of each year from baseline to 2017 is 0.953, 0.870, 0.910, 0.919, 0.939, 0.921, 0.937, and 0.899, respectively.

Abbreviations: ROC, receiver operating characteristic; DLA, deep learning algorithm; AUC, area under the receiver operating characteristic curve.

## Supplementary Table

| **Supplementary Table 1. Criteria for grading DR according to the NHS guidelines.** | |
| --- | --- |
| **Classification** | **Presence of Clinical Features** |
| R0 | Does not meet any of the following criteria |
| R1 | Microaneurysm |
|  | Retinal hemorrhage |
|  | Hard Exudate |
|  | Cotton-wool spot |
|  | Venous loops |
| R2 | Venous beading |
|  | Venous reduplication |
|  | Intraretinal microvascular abnormality |
|  | Multiple blot hemorrhages |
| R3a (active) | Neovascularization of the disc or neovascularization elsewhere in the retina |
|  | Preretinal hemorrhage or vitreous hemorrhage |
|  | Active fibrous proliferation with traction over the retina |
|  | Tractional retinal detachment |
| R3s (stable) | Evidence for peripheral retinal photocoagulation and a stable condition after treatment |
|  | Stable fibrous proliferation with or without tractional retinal detachment |
| Poor quality | Any criterion of the following: |
|  | Vessels within 1 DD of the optic disc margin or macular fovea cannot be identified |
|  | >= 50% of the area is obscured |
| Poor location | Central of the image deviates from a normal F1 or F2 image more than 2 DD |
| Abbreviations: DR, diabetic retinopathy; NHS, National Health Service; DD, disc diameter.  ^a^ Vision-threatening referable DR was defined as R2 and grades above. | |
